# RUFY, Rab and Rap Family Proteins Involved in a Regulation of Cell Polarity and Membrane Trafficking

**DOI:** 10.3390/ijms14036487

**Published:** 2013-03-21

**Authors:** Yasuko Kitagishi, Satoru Matsuda

**Affiliations:** Department of Environmental Health Science, Nara Women’s University, Kita-Uoya Nishimachi, Nara 630-8506, Japan; E-Mails: y_kitagishi@live.jp (Y.K.); smatsuda@cc.nara-wu.ac.jp (S.M.); Tel./Fax: +81-742-20-3451 (S.M.)

**Keywords:** RUFY family, RUN domain, membrane trafficking, small GTPase, NESCA, Rab, Rap

## Abstract

Cell survival, homeostasis and cell polarity rely on the control of membrane trafficking pathways. The RUN domain (comprised of the RPIP8, UNC-14, and NESCA proteins) has been suggested to be implicated in small GTPase-mediated membrane trafficking and cell polarity. Accumulating evidence supports the hypothesis that the RUN domain-containing proteins might be responsible for an interaction with a filamentous network linked to actin cytoskeleton and/or microtubules. In addition, several downstream molecules of PI3K are involved in regulation of the membrane trafficking by interacting with vesicle-associated RUN proteins such as RUFY family proteins. In this review, we summarize the background of RUN domain research with an emphasis on the interaction between RUN domain proteins including RUFY proteins (designated as RUN and FYVE domain-containing proteins) and several small GTPases with respect to the regulation of cell polarity and membrane trafficking on filamentous network.

Abbreviations

CC: Coiled Coil
FYVE: Fab-1, YGL023, Vps27, and EEA1 proteins
GAP: GTPase-activating protein
GDF: GDI displacement factor
GDI: guanine nucleotide dissociation inhibitor
GEF: guanine nucleotide-exchange factor Glut4: glucose transporter 4
PH: plekstrin homology
PIP: phosphoinositidephosphate
PIP2: phosphatidylinositol 4,5-bisphosphate
PIP3: phosphatidylinositol 3,4,5-triphosphate
PI3K: phosphatidylinositol-3 kinase
RUFY: RUN and FYVE domain-containing proteins; RUN, RPIP8, UNC-14, and NESCA proteins

## 1. Introduction

**Figure 1:**
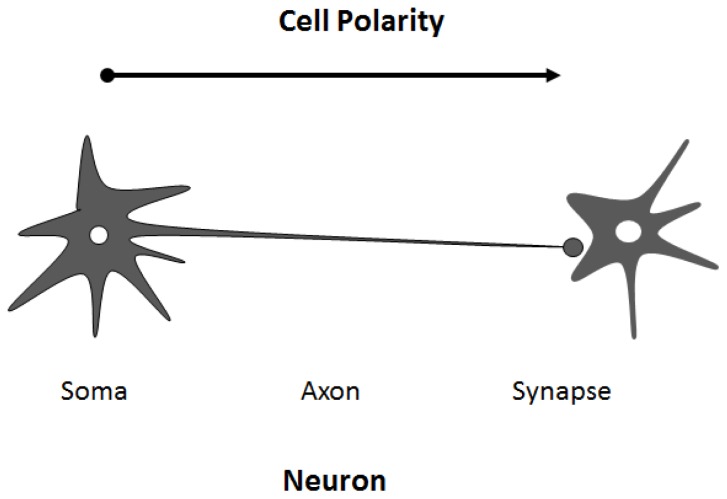
Cell polarity is observed in many cases. For example, neuronal cells are asymmetric with discrete regions responsible for different roles that may underlie the generation of specific compartments within cells, which is distinct in biochemical composition, function, and structure. An arrow indicates the direction of polarity.

**Figure 2:**
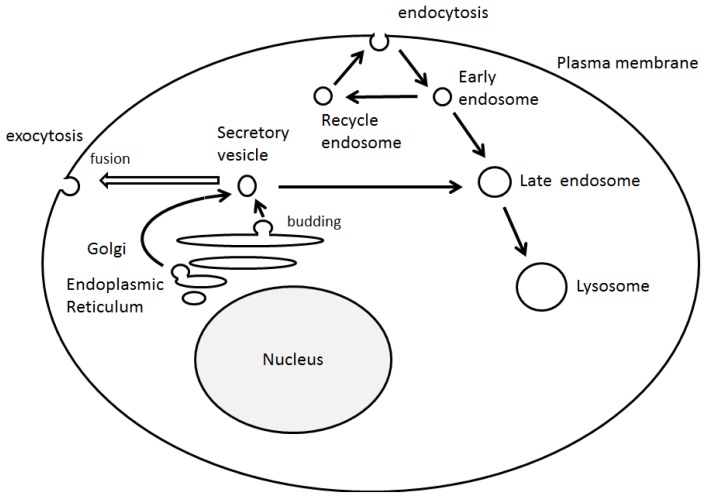
Schematic illustration of intracellular vesicle transport. The model shows several pathways used by biosynthetic secretory and internalized endocytotic cargoes to reach their destinations. Note that some critical trafficking routes have been omitted for clarity.

Asymmetric organization of cellular components and structures is called cell polarity. Establishment of the cell polarity involves many processes including signaling cascade [1], membrane trafficking events, [2] and cytoskeletal dynamics [3], which are implicated in differentiation, proliferation and morphogenesis of various cellular organisms [4]. Cell polarization is required in order for a cell to function properly. For example, the presence of axon in neuronal cells determines the directional flow of the signal (Figure 1). It is well known that initial trafficking of down-regulated protein is performed by clathrin-mediated internalization and sorting into the lumen of the endosomes [5] (Figure 2). Dysregulation of cell polarity can cause developmental disorder and cancer [6]. Studies have revealed links between cell polarity establishment and cellular membrane traffic [2,7], and the function of endosome-associated proteins has been implicated in cell polarity [8].

The direction and specificity of endosomal membrane trafficking is definitely regulated by various membrane-bound factors including protein receptors, small GTPases, and phosphoinositides [9,10]. While many components of the trafficking pathway have been ascribed, there may be additional factors that remain unknown, which precisely control the direction and specificity of membrane trafficking. In the present brief review, we summarize the function of RUN domain proteins, specifically RUFY family proteins, and their binding partners such as Rab proteins from the viewpoint of endosomal membrane trafficking. We will thus cover what is currently known about the function of protein signaling, which is also important for membrane trafficking.

## 2. RUN Domain Binds Several Signaling Molecules

The RUN domains, named after RPIP8, UNC-14, and NESCA proteins [11], might function as effectors of the small GTPase superfamily [12,13]. Because many RUN domain-containing proteins are involved in the signaling of small GTPases, including members of the Rap and Rab family, the RUN domain has been suggested to be involved in membrane trafficking [13]. The RUN domain encloses hydrophobic amino acids in the conserved positions, which is a conserved protein motif that consists of approximately 200 amino acids with binding activity to small GTP-binding proteins [11]. The sequence analysis has predicted that the RUN domain is composed of several conserved blocks, which constitute the core of a globular structure. The overall crystal structure of the RUN domain adopts a single globular fold consisting of eight alpha-helices [14]. However, it is not the only function of the RUN domain to bind a GTPase. The RUN domain binds to some molecules, including motor proteins, and the RUN domain might be responsible for an interaction with a filamentous network [13]. NESCA, a signaling adapter protein in the NGF-mediated signaling pathway, contains a RUN domain at the *N*-terminus [12]. The RUN domain of NESCA comprises nine helices, resembling the other RUN domain-containing proteins [15]. Mutational analyses have demonstrated that the RUN domain is an important structural determinant for the nuclear translocation of NESCA and that the nuclear redistribution of NESCA is essential to its neurite outgrowth-promoting properties [12]. However, the RUN domain of NESCA has different surface electrostatic distributions at the putative GTPase-interacting interface compared to the other RUN domains [15]. The RUN domain of NESCA can bind H-Ras, a downstream signaling molecule of TrkA, as well as TrkA itself, suggesting that the NESCA participates in the NGF-TrkA signaling pathway [15].

The RUN domain-containing proteins have been shown to promote endosomal fusion and are important for vesicular transport. In addition, the RUN domains appear to be required for localization to detergent-insoluble endosomal microdomains [12,13]. The physical interaction between RUN proteins and filamentous materials has been confirmed by several biochemical experiments using wild type and mutant proteins [13]. The association among small GTPases, RUN proteins, and motor proteins might reflect a novel function for these proteins in the transport of vesicular cargoes in cells. It has been reported that FYCO1 functions as an adapter linking autophagosomes to microtubule molecular motors and the Rab7, which is implicated in the phagosomal transport and fusion [16]. Kinesin-1 is a heterotetramer composed of kinesin heavy chain and kinesin light chain. UNC-14, a RUN domain protein binds to the kinesin-1 and regulates synaptic vesicle localization [17]. UNC-14 is also predicted to play an important role in multiple Ras-like GTPase signaling pathways [18]. Because RUN domains are often found in proteins involved in the regulation of Rab family small GTPases, the RUN domain has been suggested to be involved in the Rab-mediated membrane trafficking. It seems there is a common function underlying the mechanism for association of RUN domain to small GTPases and motor proteins.

## 3. Function of RUFY Family Proteins with the RUN Domain

**Figure 3:**
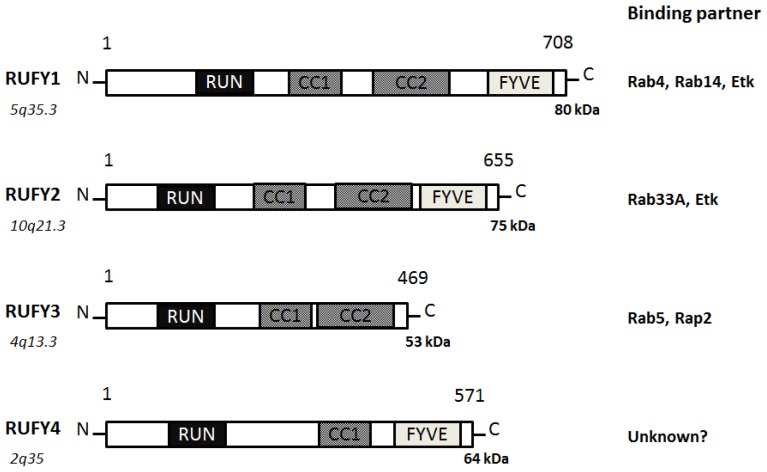
Schematic diagram indicating the domain structures of the RUFY1, RUFY2, RUFY3, and RUFY4 proteins. The functionally important sites and their interaction proteins are shown. Genomic locations of each the genes and approximate molecular mass of each the proteins are also shown at the both ends. RUN = RPIP8, UNC-14, and NESCA proteins, FYVE = Fab-1, YGL023, Vps27, and EEA1 proteins, CC = Coiled Coil.

The RUFY, designated as the RUN and FYVE domain-containing protein family, contains an amino-terminal RUN domain, and a carboxyl-terminal FYVE domain, which associate with phosphatidylinositol 3-phosphate in membranes of early endosomes [19]. Actually, RUFY proteins are localized predominantly to the early endosomes. RUFY proteins are often tyrosine-phosphorylated and the mutant lacking the phosphorylation sites fails to go to the endosomes [19]. It has been suggested Rab10, Rab11, Rab14, and RUFY proteins play an important role in the Glut4 trafficking in adipocytes and in skeletal muscle [20]. Sequence and genome analysis has revealed that a RUFY family consists of 4 members of proteins (Figure 3).

RUFY1, also known as RABIP4 or ZFYVE12, is a 708-amino acids protein that localizes to the cytoplasm and early endosome membrane [19,21]. RUFY1 has been identified as downstream effector of Etk protein kinase, which is highly expressed in testis, lung, brain and kidney. RUFY1 functions to bind PIP3-containing phospholipid vesicles and participates in early endosomal membrane trafficking [19]. The downstream effects of PI3K signaling are mediated by proteins containing a PIP3-binding module indicated by the FYVE finger domain. Many FYVE domain-containing proteins are localized at the endosomes and play an important role in endocytosis [22]. Through the SH3 and SH2 domains of Etk, the Etk interacts with RUFY1, then phosphorylates Tyr-281 and Tyr-292 of RUFY1, which is essential for the endosomal localization [19]. The Etk plays an important role in the regulation of endocytosis as a downstream effector of PI3K. Two coiled coil domains also determine endosomal localization of RUFY1 [23]. The PI3K inhibitor wortmannin blocks the endosomal localization of RUFY1 [23]. Rab14 engages in a GTP-dependent interaction with RUFY1 [24]. The active Rab14 regulates RUFY1 recruitment onto endosomal membranes, and Rab4 allows endosomal fusion [24]. The Rab14 seems to be the primary determinant of RUFY1 recruitment to the endosomes, and the FYVE domain may assist RUFY1 targeting to PIP3-enriched early endosomes [24]. Both Rab14 and RUFY1 are involved in Rab4-dependent recycling endosome, and enlargement of the early endosomes mediated by RUFY1 needs the interaction with Rab4 [25]. The RUFY1 is present in the sorting endosomes, while Rab4 is present both on sorting and recycling endosomes [26]. This would provide directional trafficking away from the recycling endosomes to the sorting endosomes. RUFY1 can also modify the kinetic parameters of Glut1 protein recycling [26].

RUFY2, also known as RABIP4R or ZFYVE13, contains a RUN domain and a carboxy terminal FYVE zinc finger, separated by two coiled coil domains [27]. Localizing to nucleus, RUFY2 is expressed in brain, lung and testis. RUFY2 as well as RUFY1 interacts with the Etk that is a tyrosine kinase involved in regulation of various cellular processes [19]. The carboxyl domain of RUFY2 binds to a negative form of Rab33A [28]. RUFY3, also known as RIPX or SINGAR1, is diffusely localized in hippocampal neurons and accumulated in the growth cones and axons [29]. RUFY3 ensures the robustness of neuronal polarity by suppressing formation of surplus axons. RUFY3 also contains the RUN domain and seems to play important roles in multiple Ras-like GTPase signaling pathways. Rab5 engages in a GTP-dependent interaction with RUFY3. RUFY3 can bind to the active Rab5 and weakly associates to Rap2 [30]. RUFY3 may function as a docking protein for distinct two small GTPases. It has been reported that oxidized LDL-containing immune complexes affect the gene expression of RUFY3 in human U937 monocytic cells [31]. RUFY4 is a 571-amino acids protein that contains a RUN domain and a FYVE zinc finger domain [32]. RUFY4 is also believed to be involved with zinc ion binding. As poorly characterized for RUFY2 and for RUFY4, little is known about the precise intracellular functions for these proteins.

## 4. Regulation of Cell Polarity and Membrane Trafficking

Cell polarity and vesicle sorting are significant processes that influence normal cell function including cell adhesion, migration, and neurotransmission. Endosomal membrane trafficking is a spatiotemporally regulated process that confirms suitable delivery of cargo via the pathway. Endosomes can bud inwardly from the membranes to form vesicles, which receive cargo from the cell surface via endocytosis and biosynthetic cargo from the late Golgi complex [33]. The endocytic trafficking has been shown to be a critical component of many signaling pathways, which is indispensable for a wide range of developmental processes [34]. In addition, the endosomal trafficking is regulated by sequential recruitment of a variety of cytosolic and membrane-bound proteins [35]. Studies have elucidated many of the key components of the events that take place during the membrane trafficking. For example, small GTPases of Rab family, its effectors, Ca^2+^ levels, and phosphoinositides may be all important. Previous studies have demonstrated that Ca^2+^ influx triggers the final step in exocytotic membrane fusion events during neurotransmission [36].

Similarly, phosphoinositides have been shown to be important for the recruitment of Rab family proteins and the binding factors [37]. Plasma membrane channels are known to be regulated by the Rab proteins [38] and phosphoinositides, which are molecules that determine the vesicular identity and direction of membrane trafficking. In particular, PIP3 is essential for the membrane trafficking of early endosome [39]. The PIP3 has lots of effector proteins in mammalian cells, all of which contain PIP3-binding motifs such as FYVE and/or PH domains [40]. Selective recruitment of these effectors by PIP3 may provide a mechanism by which the directionality for incoming vesicles and endosomes may be established. The PIP3 is thus critical for the maturation of endosomes, and for fusion events with intracellular organelles [41]. Correspondingly, disruption of PIP3 synthesis by wortmannin, a PI3K inhibitor, affects the formation of internal vesicles and the maturation of endosomes [42]. While phosphoinositides can recruit phosphoinositide-binding proteins to regulate the activity of GTPases, the GTPases can, in turn, control the activity of PIP-metabolizing enzymes.

## 5. Several Small GTPases Involved in the Membrane Trafficking

Eukaryotic cells have developed a diverse family of small GTPases to regulate the membrane trafficking pathways. Together with their effector proteins, Rab and Rap proteins mediate various aspects of vesicle formation, docking and fusion [43,44]. Some of Rab proteins recruit effectors to promote membrane fusion and vesicle formation and play a key role in early endocytic pathways. Rab proteins use the guanine nucleotide-dependent alteration mechanism to regulate the membrane traffic [45]. By binding to the guanine nucleotide exchange proteins that activate the Rab, certain effectors act to establish a feedback loop that helps to define and maintain localized domains of activated Rab proteins, which then serve to recruit the other effector molecules. Recent advances have extended the number of known Rab effectors. The Rab effectors include sorting adaptors, binding factors, lipid kinases, and lipid phosphatases, which are present on the surface of the acceptor compartment. These different tasks are carried out by a diverse collection of effector molecules that bind to specific Rab family proteins in their GTP-bound state [45]. Binding factors mediate vesicle fusion by interacting with molecules on the acceptor membrane, while lipid kinases produce phosphoinositides to define vesicular identity and the direction of membrane trafficking. In addition, the Rab cascades appear to confer directionality to membrane traffic [45].

Rab proteins are classified in specific organelle membrane proteins, establishing the basic tags to define organelle identity. Endosomal Rab proteins such as Rab 4, 5 and 9 are characterized by their localization and functional interactions with the effector proteins [46]. Rab4 and Rab11 regulate recycling of receptors from early endosomes to the cell surface via distinct pathways [47]. Rab4 functions at the level of early endosomes, and Rab11 is involved in the trafficking of cargo through recycling endosomes [48]. Rab4 is also an important player in Glut4 trafficking in adipocytes, skeletal muscle, and cardiocytes [49]. Rab5 plays important roles in clathrin-mediated endocytosis [50] and in endosome to endosome fusion. EEA1, a Rab5 effector, is recruited selectively onto early endosomes, whereas Rab5 is symmetrically distributed between the clathrin-coated vesicles and early endosomes [51]. Rab5 is localized in early endosome. In contrast, Rab7 is localized to late endosome and lysosomes [52]. Endsomal trafficking is also controlled by small GTPase Rab6, which regulates vesicle trafficking at the level of Golgi via recruitment of numerous and unrelated effectors. The crystal structure of Rab6-GTP in complex with a 378-residue internal fragment of the effector Rab6IP1 has been solved [53]. Flexibility in Rab6 mediates recognition of compositionally distinct alpha-helical coiled coils, thereby contributing to Rab6 promiscuity in effector recruitment [53]. Rab8 plays an important role in exocytic membrane trafficking from Golgi complex to plasma membrane [54]. The Rab8 is associated with myosinVI via optineurin, and the Rab8-optineurin-myosin VI complex might be involved in presenting the secretory vesicle to plasma membrane [55]. Rab9 is associated with trafficking to the Golgi network [56]. Rab11 and Rab11 family-interacting protein 3, which plays a role in membrane trafficking and regulation of actin dynamics, are both required to support the formation of filamentous virions [57]. Rab11 is also involved in recycling endosomes back to the plasma membrane. Rab14 has been implicated in phagosome and early endosome fusion. Rab14 also localizes to the Golgi complex. The Rab27A indirectly recognizes myosin Va on melanosomes via Slac2-a [58]. The Rab27B, a closely related isoform of Rab27A, is also associated with Myosin Va/VIIa via Alac2c/MyRIP [59]. Rab27 and Slac2c/MyRIP are part of a complex mediating the interaction of secretory granules with actin cytoskeleton and participate to the regulation of exocytosis. Rab35 regulates neurite-outgrowth, which is due to its direct influence on actin dynamics [60].

Rap proteins (Rap1a, -1b, -2a, and -2b) are small GTPases closely related to Ras. Rap1 is involved in various cellular processes, such as regulation of integrin-mediated cell adhesion and cadherin-mediated cell junction formation. It has been demonstrated that interaction between Rap family proteins and profilin II, an important activator of actin polymerization, is mediated via Rgl3, a RalGDS-related protein [61]. RAPL, a Rap1-associating molecule, localizes on microtubules and the activated Rap1 and RAPL control the directional migration of vascular endothelial cells [62]. Rap2 interacts with the platelet cytoskeleton by direct binding to the actin filament, which is the polymerized but not the monomeric form of actin [63]. The interaction of Rap2 with actin filaments is independent of the bound nucleotide.

Rab6 and the binding partner DENND5, a RUN domain protein, are involved in the trafficking of vesicles at the level of Golgi [64]. A RUN domain of the DENND5 plays an important role in polarized transport in Golgi through the association with sorting nexins [65], which are a large family of phosphoinositide-binding proteins that have fundamental roles in orchestrating cargo sorting [66]. In this way, small GTPases, which are related to RUN domain proteins, also associate with motor proteins and/or filamentous molecules. While small GTPases are essential for membrane trafficking, actin remodeling is the most critical. Actin is polymerized at the site of particle attachment and directs membrane extension. Many actin-associated proteins are enriched at the phagocytic cup and play important roles in phagocytosis [67]. Importantly, it is the interactions of these small GTPase proteins to the effector molecules that form a functional interaction to regulate the membrane trafficking. However, details of the biological roles elicited by effectors are largely unknown.

## 6. Perspective

Neurodegenerative diseases, including Alzheimer’s, sometimes exhibit defective endsomal trafficking. The transport would be necessary to ensure organelle homeostasis. The functional significance of membrane trafficking in the signaling pathways remains to be more established. Indeed, the RUN domain-containing proteins will be an active focus for investigation of this field. The RUFY proteins may be activated in endosomal microdomains enriched in some unidentified cytoskeletal elements. The localization of RUFY protein during the membrane trafficking seems to be dynamic. Although specific kinetic information would be required, RUFY and Rab proteins may have an inhibitory function in endocytosis, which is similar to the case of Rab15 in neuronal system [68]. This inhibitory effect could result from a negative regulation of endosome recycling. Likewise, RUFY3 may ensure the strength of neuronal polarity by suppressing formation of surplus axons. The molecular details of how RUFY3 inhibits the formation of surplus axons remain important issues for future investigation [29,69]. Deciphering the precise mechanisms will provide new insight into the physiological roles of these interesting molecules in regulating membrane traffic [13]. It is expected that future studies will address these issues in order to achiee a better understanding of the potential partners and roles of the RUFY and Rab/Rap proteins.
